# Loss of the Polyketide Synthase StlB Results in Stalk Cell Overproduction in *Polysphondylium violaceum*

**DOI:** 10.1093/gbe/evaa079

**Published:** 2020-04-18

**Authors:** Takaaki B Narita, Yoshinori Kawabe, Koryu Kin, Richard A Gibbs, Adam Kuspa, Donna M Muzny, Stephen Richards, Joan E Strassmann, Richard Sucgang, Kim C Worley, Pauline Schaap

**Affiliations:** e1 School of Life Sciences, University of Dundee, United Kingdom; e2 Department of Molecular and Human Genetics, Baylor College of Medicine, Houston, Texas; e3 Verna and Marrs McLean Department of Biochemistry and Molecular Biology, Baylor College of Medicine, Houston, Texas; e4 Department of Biology, Washington University; e5 Department of Life Science, Faculty of Advanced Engineering, Chiba Institute of Technology, Chiba, Japan; e6 The Welch Foundation, Houston, TX; e7 Genome Sequencing Center, University of California Davis, Davis, CA

**Keywords:** *Polysphondylium violaceum* genome, cell type-specific transcriptome, evolution of novel cell types, genetic transformation, DIF-1, polyketide synthase

## Abstract

Major phenotypic innovations in social amoeba evolution occurred at the transition between the Polysphondylia and group 4 Dictyostelia, which comprise the model organism *Dictyostelium discoideum*, such as the formation of a new structure, the basal disk. Basal disk differentiation and robust stalk formation require the morphogen DIF-1, synthesized by the polyketide synthase StlB, the des-methyl-DIF-1 methyltransferase DmtA, and the chlorinase ChlA, which are conserved throughout Dictyostelia. To understand how the basal disk and other innovations evolved in group 4, we sequenced and annotated the *Polysphondylium violaceum* (*Pvio*) genome, performed cell type-specific transcriptomics to identify cell-type marker genes, and developed transformation and gene knock-out procedures for *Pvio.* We used the novel methods to delete the *Pvio stlB* gene. The *Pvio stlB^−^* mutants formed misshapen curly sorogens with thick and irregular stalks. As fruiting body formation continued, the upper stalks became more regular, but structures contained 40% less spores. The *stlB^−^* sorogens overexpressed a stalk gene and underexpressed a (pre)spore gene. Normal fruiting body formation and sporulation were restored in *Pvio stlB^−^* by including DIF-1 in the supporting agar. These data indicate that, although conserved, stlB and its product(s) acquired both a novel role in the group 4 Dictyostelia and a role opposite to that in its sister group.

## Introduction

Multicellularity allowed cells to specialize into roles that support the propagation of others within the organism. Modern plants and animals owe their extensive morphological complexity to the progressive specialization of such somatic cells. Unraveling how this occurred is crucial to understand the mechanistic foundation of all extant developmental programs. Dictyostelia represent a group of multicellular organisms, which, depending on the species, contain only spores to propagate the species, or additionally contain cells that form a stalk and other cell types to support the spore mass. Dictyostelia can be subdivided into four major and some minor groupings, with a minor group containing *Polysphondylium violaceum* (*Pvio*) as sister clade to major group 4, which contains *Dictyostelium discoideum* (*Ddis*) and all other species that use cAMP as chemoattractant (Schaap et al. 2006; Romeralo et al. 2013; Schilde et al. 2019). Apart from the use of cAMP, group 4 shows other innovations, such as proportion regulation of prespore and prestalk cells and acquisition of three new cell types, which form a basal disk that supports the stalk and upper- and lower cups that cradle the spore mass (Schilde et al. 2014). DIF-1 was identified >30 years ago as a secreted factor that induces stalk cell differentiation in *Ddis* in vitro (Morris et al. 1987). Its biosynthetic enzymes were identified more recently and are the polyketide synthase StlB (Saito et al. 2008), the des-methyl-DIF-1 methyltransferase, DmtA (Thompson and Kay 2000), and the flavin-dependent halogenase, ChlA (Neumann et al. 2010). Null mutants in either of these mutants form weaker migrating slugs with a reduced number of prestalk cells and fruiting bodies that lack the basal disk, which consists of stalk-like cells. Because the stalk is still formed in all null mutants in DIF-1 synthetic enzymes, it was concluded that DIF-1 is mainly required for basal disk, rather than for stalk cell differentiation (Saito et al. 2008). A DIF-1 dechlorinase, DrcA, which deactivates DIF, was also identified, but *drcA^−^* mutants showed no developmental defects (Velazquez et al. 2011). Recent studies showed that polyketides produced by another polyketide synthase, StlA, may cooperate with DIF-1 in inducing prestalk differentiation, because a *stlB^−^/stlA^−^* mutant showed reduced expression from the *pstA* region of the *ecmA* prestalk promoter (Sato et al. 2013, 2016).

Except for one clade, the Acytostelids, all Dictyostelids form a cellular stalk, but the basal disk appears to be restricted to group 4 and one species, *Dictyostelium mexicanum*, in group 1 (Romeralo et al. 2013). Nevertheless, the three DIF-1 biosynthetic enzymes StlB, DmtA, and ChlA are present in all four *Dictyostelium* taxon groups (Gloeckner et al. 2016). Interestingly, in the Acytostelid *Acytostelium subglobosum*, the function of at least one of the enzymes, DmtA, is not conserved, because *A. subglobosum dmtA* cannot restore the disk-less phenotype of a *Ddis dmtA^−^* mutant (Mohri et al. 2014). Biochemical analysis of DIF-1 metabolism in other species than *Ddis* suggests that another group 4 species, *Dictyostelium mucoroides*, as well as *Pvio* could synthesize DIF-1, but this was not the case for *Dictyostelium vinaceo-fuscum* in group 3, although this species also produces chlorinated compounds (Kay et al. 1992). *Pvio* is not reported to form a basal disk, but owes its name to the fact that apart from the main stalk, its fruiting bodies have regular whorls of side branches, each consisting of a stalk and spore head.

We investigate the molecular changes that caused the emergence of novel cell types in the course of evolution. Investigation of *Pvio* is critical to understand this process in the group 4 *Dictyostelia*, but thus far there were no protocols to genetically transform this *Pvio*. We therefore developed protocols for transformation, gene knock-out, and overexpression for this species, analyzed the transcriptomes of its individual cell types and performed transcriptome-guided gene model prediction of an existing *Pvio* draft genome. To investigate a possible role for DIF-1 in *Pvio*, we deleted its *stlB* gene, which is required for the first step in DIF-1 biosynthesis. Surprisingly, the *Pvio stlB^−^* mutant showed an increase rather than a decrease in stalk-like cells, suggesting that the role of DIF-1 as inducer of this cell type emerged relatively recently in group 4.

## Materials and Methods

### *Pvio* Genome Sequencing and Transcriptomics

The genome of *Pvio QSvi11* was sequenced using the 454 sequencing technology (Voelkerding et al. 2009). Sequencing libraries were prepared, sequenced, and assembled as described previously (Ostrowski et al. 2015). Data from the genome sequence are available under BioProject PRJNA45881 in the ENA and NCBI.

### *Pvio* Cell Type-Specific Transcriptomes and Genome Annotation

Total RNA was prepared from purified *Pvio QSvi11* spore and stalk cells as well as vegetative cells from *Pvio*, obtained from three separate experiments, following the cell isolation protocol previously described (Kin et al. 2018). The qualities of the RNAs isolated in three independent experiments were assessed with TapeStation (Agilent) to be excellent (RIN > 8.5) and cDNA libraries were prepared using the Truseq Stranded mRNA Library Prep Kit (Illumina). About 75-bp paired-end reads were sequenced with Illumina NextSeq 500 at the Tayside Centre for Genomic Analysis (https://tcga.org.uk; last accessed April 21, 2020) in two independent runs and were archived in ENA as Bioproject PRJEB34080. The RNA-Seq data were used to annotate the *Pvio QSvi11* genome (NCBI Bioproject PRJNA45881) using the pipeline BRAKER1 (Hoff et al. 2016). BRAKER1 utilizes two separate programs, GeneMark-ET (Lomsadze et al. 2014) and AUGUSTUS (Stanke et al. 2008), to create gene models. More specifically, GeneMark-ET incorporates unassembled RNA-Seq reads and generates ab initio gene predications. A subset of GeneMark-ET predicted genes was then used to train AUGUSTUS, which generated the final gene models.

To obtain cell type-specific gene expression profiles, a software package RSEM (Li and Dewey 2011) was used to estimate transcript abundances, with the Bowtie2 aligner for mapping RNA-Seq reads to gene features. We used the “-estimate-rspd” option in RSEM to estimate read start position distributions, which is expected to facilitate more accurate abundance estimates for 3′ biased reads produced from oligo-dT primed libraries (Li and Dewey 2011). The expression data in transcripts per million are listed in supplementary file Pvio_Celltype_TPM.xlsx, Supplementary Material online.

### *Pvio* Transformation

*Pvio QSvi11* was cultured on 1/5th SM agar (Formedium, United Kingdom) in association with *Escherichia coli* 281. When plates started to clear, cells were harvested, washed with KK2 (20 mM K-phosphate, pH 6.2), and incubated in culture dishes at 2×10^5^ cells/ml in HL5 overnight. Cells were harvested and washed with H-50 buffer (Pang et al. 1999) and resuspended in H-50 buffer at 5×10^7^ cells/ml. An aliquot of 0.1 ml of the cell suspension was mixed with 10 µg of plasmid vector DNA, transferred to a 1-mm gap electroporation cuvette, and electroporated by Gene Pulser II (BIORAD) at 0.65 kV and 25 µF with two pulses. The cells were immediately transferred to a dish containing 10 ml HL5 medium and allowed to recover at 21 °C for 5–6 h. The cells were resuspended in 10 ml KK2, containing autoclaved *Klebsiella aerogenes* (*K.aer*) (final OD_600_=8.5), 10% HL5, and 20 µg/ml of G418, shaken for 48 h at 150 rpm, and distributed with G418-resistant *E. coli* over 1/5th SM agar, containing 50 µg/ml of G418. G418-resistant *E. coli* were obtained by transforming *E. coli* B/r with pUC4K (Addgene), which harbors a kanamycin/neomycin selection gene. Transformants were picked up after 4 days and were further grown and developed on 1/5th SM agar with *E. coli*.

### Plasmid Constructs and Knock-Out Diagnosis

The *Pvio* ortholog of *Ddis stlB* was identified by BlastP search of the assembled and translated *Pvio* QSvi11 transcripts with the *Ddis* StlB sequence, whereas the genome sequence was retrieved by TBlastN search of the Pvio QSvi11 whole-genome shotgun sequence (Bioproject PRJNA45881) with *Ddis* StlB. Two *stlB* fragments of 1,606 bp (fragment 1) and 1,339 bp (fragment 2) were amplified from *Pvio* QSvi11 genomic DNA, using primer combinations Pv_stlB_Frag1F/Pv_stlB_Frag1R and Pv_stlB_Frag2F/Pv_stlB_Frag2R (supplementary table S1, Supplementary Material online), respectively, and cloned into vector pLoxP-NeoIII (Kawabe et al. 2012) to flank the loxP-Neo selection cassette. The resulting pPvStlB-KO construct was excised with *Not*I/*Xho*I and transformed into *Pvio* QSvi11. Genomic DNAs were isolated from G418-resistant clones and probed by PCR with primer pairs Pv_stlB_NegF/Pv_stlB_NegR and Pv_stlB_Pos5′/cas1 for *stlB* gene disruption by homologous recombination (supplementary fig. S2, Supplementary Material online). From 27 investigated clones, 19 clones showed insertion of the LoxP-Neo cassette in *stlB*. To remove the Neo cassette from the *Pvio stlB^−^* cells, cells were transformed with pA15NLS.Cre (Faix et al. 2004) for transient expression of Cre recombinase. Transformed clones were replica-plated with G418-resistant *E. coli* on lactose/peptone agar supplemented with 50 µg/ml G418 for negative selection.

To generate a fusion of the *LacZ* reporter with (pre)stalk gene promoters, putative orthologs of the *Ddis* (pre)stalk genes *ecmA* or *ecmB* were identified by BlastP of the translated *Pvio* QSvi11 transcriptome. Phylogenetic inference of the retrieved sequences revealed that *ecmA* and *ecmB* are group 4-specific gene duplicates and that their common ancestor was duplicated in *Pvio* (supplementary fig. S3, Supplementary Material online). The *Pvio* genes were named *ecmAB1* and *ecmAB2*. The 868-bp 5′ region of *ecmAB1*, which includes the start codon, was amplified from *Pvio* gDNA using primer pair Pv_ecmAB1_P F/Pv_ecmAB1_P R (supplementary table S1, Supplementary Material online) and, after *Xba*I/*Bgl*II digestion, cloned into the XbaI and BglII sites of pDdGal-17(H-) (Harwood and Drury 1990) to yield Pv_ecmAB1-lacZ. Both plasmids were transformed into *Pvio* wild-type cells or G418 sensitive *stlB^−^* cells (see above) and after recovery in HL5, transformants were clonally selected during coculture with G418-resistant *E. coli* on 1/5th SM agar, containing 50 µg/ml of G418.

### B-Galactosidase Histochemistry

*Pvio* cells transformed with Pv-ecmAB1-gal were harvested from growth plates, distributed at 10^6^ cells/cm^2^ on nitrocellulose filters supported by nonnutrient (NN) agar (1.5% agar in 8.8 mM KH_2_PO_4_ and 2.7 mM Na_2_HPO_4_) and incubated at 22 °C until the desired developmental stages had been reached. Filters with developing structures were next fixed in glutaraldehyde and stained with X-gal as previously described (Dingermann et al. 1989).

### Quantitative Reverse Transcription-Polymerase Chain Reaction

Wild-type and *stlB^−^* cells were distributed over NN agar at 10^5^ cells/cm^2^ and incubated at 4 °C overnight. Cells were next incubated at 22 °C and RNA was isolated from the cells at 3 h intervals. cDNAs were synthesized from 1 µg RNA with the SensiFAST cDNA synthesis kit (Bioline, United Kingdom), which generates 20 µl aliquots of cDNA. cDNA samples of 1 μl were used for quantitative reverse transcription-polymerase chain reaction (RT-qPCR) with PerfeCTa SYBR Green SuperMix (QuantaBio) and analyzed by LightCycler 96 System (Roche, Germany). The *Pvio* stalk gene *ecmAB1*, the (pre)spore gene *g1612*, and the constitutively expressed gene *rpb5* were amplified with primer pairs Pv-ecmAB1-51/Pv-ecmAB1-31, Pv-g1612-51/Pv-g1612-31, and Pv-rpb5-51/Pv-rpb5-31 (supplementary table S1, Supplementary Material online). The PCR program consisted of 45 cycles, with 30 s at 95, 55, and 72 °C each.

## Results

### *Polysphondylium violaceum* Genome Sequencing and Gene Model Annotation

The genome of *Pvio* Qsvi11 was sequenced using the Roche 454 sequencing platform, assembled, and archived in NCBI by some of us (J.E.S., A.K., R.S., D.M.M., S.R., K.C.W., and R.A.G.) in 2012. The others collected RNA-Seq data of spore, stalk, and vegetative cells from *Pvio QSvi11* to identify conserved cell type-specific genes and used the data to perform gene model prediction of the archived genome. The RNA-Seq reads and the whole-genome assembly were processed with the pipeline BRAKER1 (Hoff et al. 2016), which combines two genome annotations tools, GeneMark-ET and AUGUSTUS. The final gene models generated by AUGUSTUS yielded a total of 10,597 coding sequences (table 1). The *Pvio* genome is at 25.7 Mb only somewhat larger than that of *Dictyostelium lacteum* (*Dlac*), thus far the smallest published dictyostelid genome. This is reflected in the number of coding sequences also being relatively small. With a 68.2% A/T content, the codon bias of *Pvio* is more similar to that of the groups 1, 2, and 3 representative species *Dictyostelium fasciculatum* (*Dfas*), *Polysphondylium pallidum* (*Ppal*), and *Dlac* than to that of *Dictyostelium purpureum* (*Dpur*) and *Ddis* in its sister group 4.

**Table 1:** Features of Published *Dictyostelid* Genomes

	*Ddis*	*Dpur*	*Pvio*	*Dlac*	*Ppal*	*Dfas*
Contigs/supercontigs	226/6	1,213/799	1,228	54/54	52/41	33/25
Total nucleotides (Mb)	33.9	33.0	25.7	23.4	32.9	31.0
Average contig length (kb)	155	27	21	432	320	1,064
A/T content (%) overall/in CDS	77.6/72.6	75.5/ND	68.2/65.5	70.2/67.8	68/63.8	66.2/63.2
Coding sequences (CDS)	13,258	12,410	10,597	10,232	11,694	12,007
Average gene length	1,604	1,760	1,624	1,712	1,634	1,696
Gene density (CDS per Mb)	396	376	412	437	375	392
DNA in CDS (Mb)	21.3	21.8	17.3	17.5	18.7	20.2
Intergenic DNA (Mb)	12.6	11.2	8.4	5.8	14.2	10.8

Note.—Genome statistics for *Ddis*, *Dpur, Dlac, Ppal*, and *Dfas* were retrieved from Eichinger et al. (2005), Gloeckner et al. (2016), Sucgang et al. (2011), and Heidel et al. (2011), respectively. ND, not determined. *Dlac*, *Ppal*, and *Dfas* were renamed as *Tieghemostelium lacteum*, *Heterostelium album*, and *Cavenderia fasciculata* in a new classification of Dictyostelia (Sheikh et al. 2018), which may need revision in the near future (Schilde et al. 2019) and is therefore not used here.

### Transformation of *Pvio*

To establish genetic transformation of *Pvio*, we first tested resistance to the antibiotics G418, hygromycin, and blasticidin that are routinely used to select *Ddis* transformants. Many other *Dictyostelium* species show high general resistance to antibiotics, rendering selection of transformed wild-type cells problematic. Proliferation of *Pvio QSvi11* grown in suspension with heat-killed *K.aer* was suppressed by 20 µg/ml G418 or 30 µg/ml hygromycin, indicating that G418 and hygromycin can potentially be used for selection of transformed cells. To test plasmid entry into the cells, we electroporated *Pvio* with the *LacZ* expression vector pA15-Gal (Harwood and Drury 1990) using a protocol developed for *Ppal* (Kawabe et al. 1999), then incubated cells for 5 h in HL5 and measured β-galactosidase activity in freeze-thawed lysates. The extracts from plasmid-treated cells showed 4.9-fold higher β-galactosidase activity than mock-electroporated cells, indicating that the pA15-Gal had entered *Pvio*. We also examined the transient expression of *LacZ* from pA15-Gal after electroporation of other species, such as *D. mucoroides* S28b, *Dictyostelium aureo-stipes* YA6, *D*. *fasciculatum* SH3, and *Dlac* TK, but did not detect any increased β-galactosidase activity.

After transformation, *Pvio* cells were incubated with heat-killed *K.aer* in suspension culture with 20 µg/ml G418 for 1 week and then distributed with heat-killed *K.aer* over NN agar plates containing 20 µg/ml G418. After several days, a few clones developed. PCR analysis showed that one of six tested clones contained the NeoR cassette from pA15-Gal, indicating that stable transformation is possible in *Pvio* (supplementary fig. S1*A*, Supplementary Material online). Southern-blot analysis showed that only a single copy of the vector was inserted in the genome (supplementary fig. S1*B*, Supplementary Material online). When needed, this facilitates recycling of the G418-resistance cassette using the cre-loxP system (see below). Although many nontransformed cells were also recovered after selection, we improved the procedure by prolonged selection with higher G418 concentrations and other incremental changes, and our current standard selection procedure (see Materials and Methods) now mostly yields transformed cells. *Pvio* transformation was also achieved by using vectors which contain the hygromycin-resistance cassette (unpublished results).

### Knock-Out of *stlB in Pvio*

**Fig. 1.**
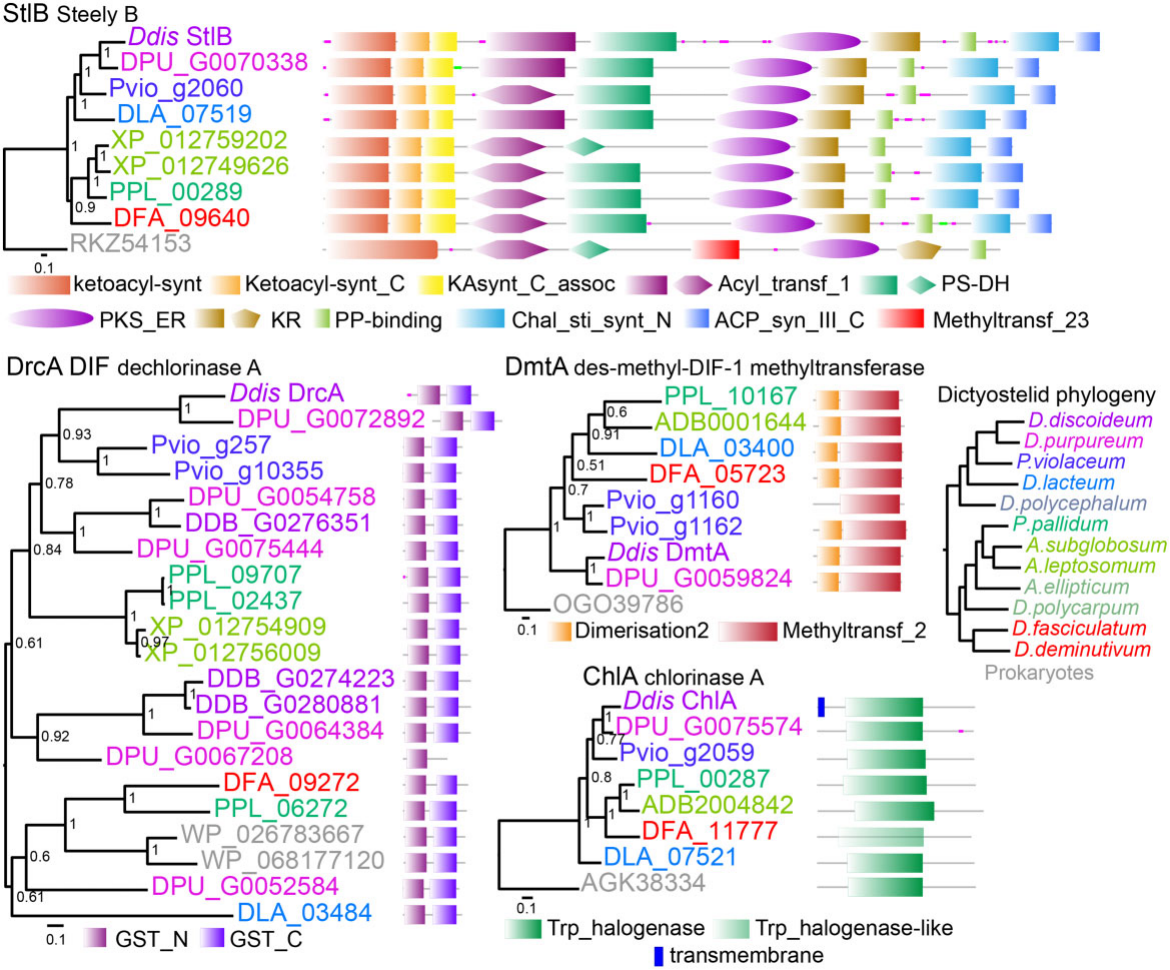
—Conservation of DIF-1 metabolic enzymes across Dictyostelia. Putative orthologs of the DIF-1 metabolic enzymes StlB, DmtA, ChlA and DrcA were retrieved by BlastP search of social ameba genomes and GenBank. Sequences were aligned using Clustal Omega (Sievers and Higgins 2014) with five combined iterations and phylogenetic trees were inferred with MrBayes (Ronquist and Huelsenbeck 2003) using a mixed amino acid substitution model with rate variation between sites estimated by a gamma distribution. Bayesian posterior probabilities of the tree nodes are indicated and trees are annotated with the functional domain architecture of the proteins as analyzed by SMART (Schultz et al. 1998). Domains present at E-values above threshold are shown in wash-out color. Gene IDs and locus tags are color coded to reflect the host species as indicated in the dictyostelid core phylogeny (top left), retrieved from Singh et al. (2016). Note that single-gene phylogenies may not strictly follow the species phylogeny, which is based on 47 genes, due to limited phylogenetic signal or clade-specific gene gain and loss.

We are interested in the emergence of novel somatic cells during the evolution of multicellularity. In Dictyostelia, this occurs particularly in group 4 with its unique basal disk cells to support the stalk and upper and lower cup cells to anchor the spore mass to the stalk. Basal disk differentiation is induced by the chlorinated polyketide DIF-1 (Saito et al. 2008), which is synthesized by the polyketide synthaste StlB and further modified by DmtA and ChlA (Thompson and Kay 2000; Saito et al. 2008; Neumann et al. 2010), enzymes that are conserved throughout Dictyostelia (Gloeckner et al. 2016). To investigate the ancestral role of DIF-1 signaling, we attempted to delete the *Pvio stlB* gene. DNA sequences for *stlB* and the other DIF-1 metabolizing enzymes were retrieved from *Pvio* QSvi11 transcriptome and genome sequences as highest scoring bidirectional BLAST hits. Gene orthology was further confirmed by phylogenetic inference (fig. 1).

**Fig. 2.**
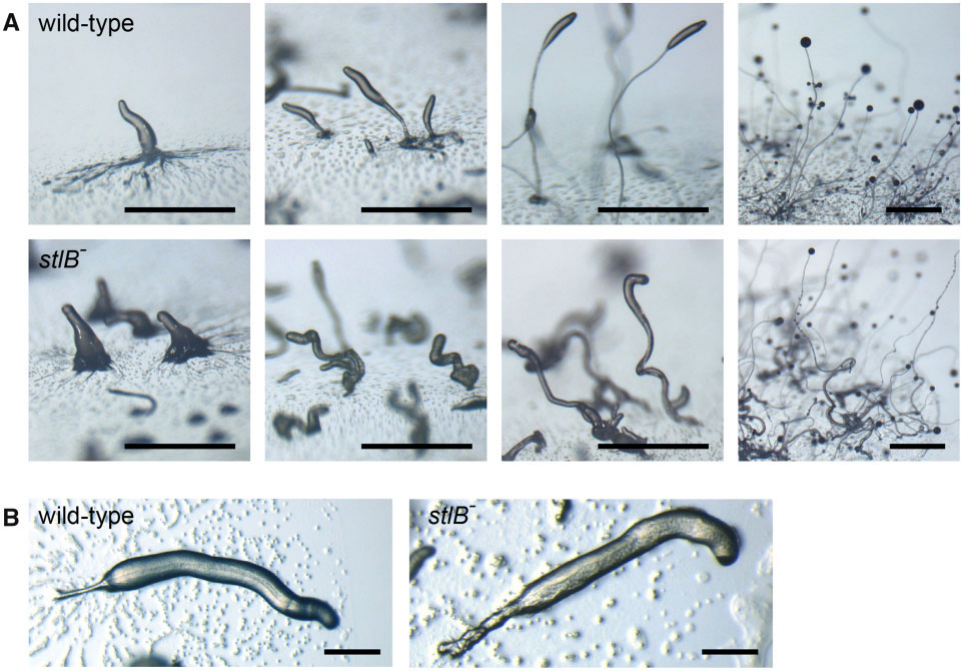
—Phenotype of the *Pvio stlB^−^* mutant. *Pvio* wild-type and *stlB^−^* cells were incubated overnight at 4 °C on NN agar at 10^6^ cells/cm^2^ and then transferred to 22 °C. (*A*) Developmental morphologies were sequentially photographed at 5, 8, 10, and 30 h at 22 °C. Bars: 1 mm. (*B*) Stalk formation after 5–8 h at 22 °C, imaged at higher magnification. Bars: 0.2 mm.

A *stlB* knock-out construct was designed to replace the essential phospho-pantetheine binding domain of polyketide synthases like *stlB* (Moretto et al. 2017) with the G418-resistance cassette and stop codons in all reading frames. Homologous recombination was extremely efficient, because 70% of the G418-resistant clones proved to be knock-outs (supplementary fig. S2, Supplementary Material online). The knock-out clones aggregated normally, but formed abnormal primary sorogens, which were overall thicker than those of wild-type and had a curly appearance (fig. 2*A*).

*Pvio* cell masses are normally lifted up rapidly after aggregation by the newly formed stalk, which penetrates the cell mass. This process was delayed in the *stlB^−^* mutants and the early stalk was irregularly shaped and several cells thick (fig. 2*B*), compared to the mostly single cell width of *Pvio* wild-type stalks. As fruiting body formation progressed, stalks became thinner and looked more regular. The *stlB^−^* fruiting bodies also appeared to form fewer side branches, and those that formed often lacked stalks. However, compared with other wild-type *Pvio* strains, which robustly form many regular whorls of side branches, strain QSvi11, which was chosen here because of its sensitivity to antibiotics, shows rather variable branching, with many fruiting bodies showing no branches at all.

### Stalk Gene Expression in *Pvio stlB^−^* Mutants

**Fig. 3.**
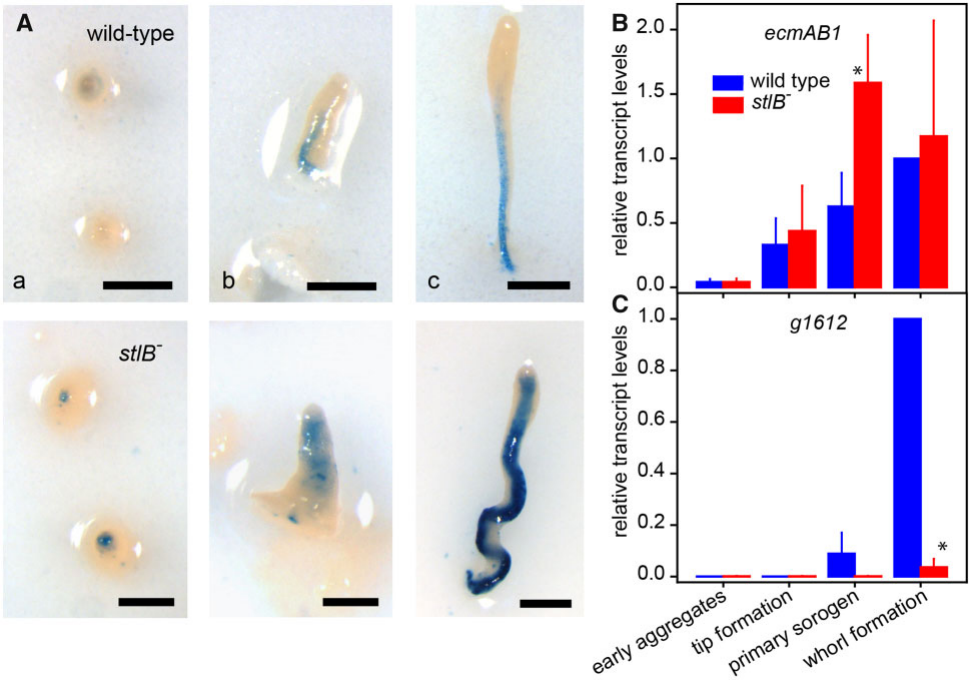
—Stalk and spore gene expression in *Pvio stlB^−^*. (*A*) EcmAB1 expression pattern. *Pvio* wild-type and *stlB^−^* cells, transformed with the Pv_ecmAB1-lacZ vector, were incubated on nitrocellulose filters supported by NN agar until they had reached the tipped mound (a) and early (b) and late (c) primary sorogen stage. Structures were then fixed and stained with X-gal. Bars: 0.2 mm. (*B*) Stalk and spore transcript levels. *Pvio* wild-type and *stlB^−^* cells were incubated on NN agar at 4 °C overnight and then at 22 °C for 3, 6, 9, and 12 h, when they had reached the indicated stages. Total RNA was isolated and levels of *ecmAB1* stalk-specific transcripts, *g1612* (pre)spore-specific transcripts, and *rpb5* constitutively expressed transcripts were determined by RT-qPCR. Data for *ecmAB1* and *g1612* were normalized to *rpb5* transcript levels and expressed relative to transcript levels at 12 h for wild-type cells. Means and SD of three experiments, assayed with three technical replicates each, are presented. Asterisks indicate significant differences between wild-type and *stlB^−^* transcript levels (*t*-test, *P*<0.05).

To investigate whether the *Pvio stlB^−^* mutant expressed known DIF-regulated stalk genes, we searched for *ecmA* and *ecmB* orthologs in the *Pvio* genome. Two close *Pvio* relatives to *ecmA* and/or *ecmB* were detected, which form a sister clade to *Ddis ecmA* and *ecmB* (supplementary fig. S3, Supplementary Material online). They are therefore not true orthologs of either, but the result of an independent gene duplication of the ancestor to *Ddis ecmA* and *ecmB.* We therefore named the *Pvio* genes *ecmAB1* and *ecmAB2.* We prepared fusion constructs of the *LacZ* reporter gene and the available 868-bp promoter region of the *ecmAB1 gene.* The loxP neomycin cassette was excised with cre-recombinase from the disrupted *stlB* gene by transformation of the *stlB^−^* mutant with pA15NLS.Cre (Faix et al. 2004), and a G418 sensitive *stlB^−^* clone as well as wild-type *Pvio* were transformed with the Pv_ecmAB1-lacZ construct. Figure 3*A* shows that in wild-type structures *Pvio ecmAB1* is weakly expressed in aggregate tips and more strongly in the stalk of early and later sorogens at some distance from the tip. In the *stlB^−^* mutant, aggregate tips express *ecmAB1* more strongly than is the case in wild-type, whereas both early and late sorogens express *ecmAB1* in the stalk starting from just below the tip. Particularly, in the later sorogens, *ecmAB1* was expressed very strongly and conform to the appearance of the sorogens under transillumination (fig. 2*B*), the *ecmAB1-*stained stalk was thicker than that of wild-type as well as curly.

The intensity of LacZ staining in transformed cells may reflect plasmid copy number rather than the level of promoter activity. We therefore also measured *ecmAB1* transcript levels in the developing sorogens directly by RT-qPCR. Figure 3*B* shows that *ecmAB1* transcripts are significantly higher in *stlB^−^* mutants than in wild-type cells. To investigate whether the increase in stalk gene expression is accompanied by a loss in prespore gene expression, we also sought for homologs of *Ddis* spore coat genes in *Pvio.* Twelve homologs were detected (supplementary fig. S4, Supplementary Material online), but orthology with *Ddis* spore coat genes could not be unequivocally established.

We used RNA-Seq data of purified *Pvio* spores, stalks, and vegetative cells (supplementary file Pvio_Celltype_TPM.xlsx, Supplementary Material online) to assess which genes were specifically expressed in spores (supplementary table S2, Supplementary Material online). This only appeared to be the case for *g1612* and its close homolog *g7962*. We therefore used *g1612* as a (pre)spore marker. RT-qPCR with *g1612*-specific primers showed that in wild-type *g1612* transcript levels first appeared in primary sorogens and strongly increased when sorogens formed the first whorls of side branches with mature spores. Expression of *g1612* was much reduced in *stlB^−^* cells (fig. 3*B*), which could result from overproduction of stalk cells at the expense of prespore cells or from the lack of side branch formation in the *stlB^−^* sorogens.

In conclusion, unlike *Ddis*, where deletion of *stlB* results in fruiting bodies with thinner stalks and lacking the basal disk with its stalk-like cells, the *Pvio stlB^−^* mutant actually overproduces stalk cells, accompanied by overexpression of at least one stalk gene and underexpression of a (pre)spore gene.

### Effects of DIF-1 on the *Pvio stlB^−^* Mutant

**Fig. 4.**
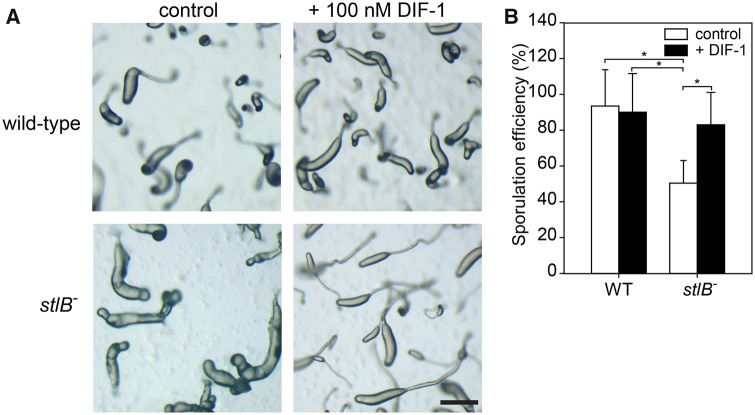
—Effects of DIF-1 on the *stlB*^−^ phenotype. (*A*) Development. *Pvio* wild-type and *stlB^−^* cells were plated on NN agar with or without 100 nM DIF-1, incubated at 4 °C overnight, and then for 6 h at 22 °C. Bar: 0.2 mm. (*B*) Sporulation efficiency. Cells were developed on 1 cm^2^ pieces of filter paper supported by NN agar with or without 100 nM DIF-1. After 2 days, filters with mature fruiting bodies were vortexed vigorously in 1 ml of KK2 and spores were counted. Sporulation efficiency was calculated by dividing the number of spores counted by the total number of cells plated initially. Mean and SD of three experiments performed in duplicate are shown. Significant differences are indicated by asterisks (*t*-test, *P*<0.02).

Normal slug and fruiting body formation are restored when the *Ddis stlB^−^* mutant is allowed to develop on agar that contains 100 nM DIF-1. We examined whether this is also the case for the *Pvio stlB^−^* mutant. Figure 4*A* shows that 100 nM DIF-1 has no effect on sorogen formation by wild-type *Pvio*. However, the abnormal sorogens of *Pvio stlB^−^* are fully restored to normality in the presence of 100 nM DIF-1. We also tested whether DIF-1 affects sporulation in *Pvio stlB^−^*. Figure 4*B* shows that ∼90% of wild-type cells develop into spores both in the absence and in the presence of DIF-1. For the *stlB^−^* mutant, this is only 50% in the absence of DIF-1 and 80% in its presence. These experiments show that DIF-1 restores normal stalk formation and morphogenesis as well as sporulation in the *stlB^−^* mutant, demonstrating that the developmental defect caused by the *stlB* lesion is likely due to defective polyketide synthesis.

## Discussion

### *Polysphondylium violaceum* Can Be Genetically Transformed

In the course of our evolutionary comparative studies, we have attempted to make a range of species amenable to genetic transformation. The major obstruction for doing so is the extreme resistance of many species to antibiotics like G418, blasticidin, hygromycin, and nourseothricin that are used for transformation of the model species *Ddis.* Even *Ppal*, which we have transformed routinely for many years, is completely resistant to blasticidin and hygromycin and is only effectively killed by 300 µg/ml G418 as opposed to 10 µg/ml for *Ddis*. In addition, species that are sensitive to antibiotics can resist uptake of plasmid DNA by electroporation or by transfection with calcium phosphate-based transformation protocols. *Pvio* QSvi11 is sensitive to G418 and hygromycin, but the developmentally more robust strain *Pvio* P6 is again highly resistant.

We here show that *Pvio QSvi11* can be transformed and that by using the cre-lox system the selectable marker can be recycled. An added bonus of this strain is that homologous recombination is extremely efficient. Including *stlB*, we have at present deleted four genes in *Pvio*, with all attempts yielding at least 70% positive clones. The position of *Pvio* as sister clade to group 4 makes it particularly suitable for investigating the genetic change that caused the immense phenotypic innovations of group 4.

### Conservation of DIF-1 Metabolizing Enzymes

The polyketide synthase StlB is well conserved throughout the dictyostelid phylogeny, inclusive of its elaborate domain architecture (fig. 1), suggesting that synthesis of the polyketide backbone of DIF-1 should be possible for all species. The des-methyl-DIF-1 methyltransferase DmtA and single chlorinating enzyme ChlA are also conserved as orthologs, albeit that in the group 1 species *Dfas* the halogenase domain of ChlA is no longer recognized by the domain Markov model, and the DmtA of the group 2 species *A. subglobosum* is not functionally equivalent to *Ddis* DmtA (Mohri et al. 2014). Chlorinated compounds are made by all tested species, but only those in group 4 and probably *Pvio* synthesize DIF-1 (Kay et al. 1992). The dechlorinating enzyme DrcA is one of many gluthatione-S-transferases and is the major enzyme that inactivates DIF-1 in group 4 (Velazquez et al. 2011). The DrcAs from group 4 species cluster in a separate clade (fig. 1), whereas the closest nongroup 4 homologs are about equidistant between DrcA and other group 4 gluthatione-S-transferases. Combined with observations that *Pvio* cannot dechlorinate DIF-1 (Van Es et al. 1995), this suggests that the ability to dechlorinate DIF-1 evolved only in group 4.

### Loss of StlB in *Pvio* Results in Excessive Stalk Cell Differentiation

In *Ddis*, deletion of *stlB* results in longer weaker slugs and fruiting bodies with thinner stalks that lack the basal disk (Saito et al. 2008). The basal disk is a group 4 innovation and is not present in its sister species *Pvio* (Schilde et al. 2014). To investigate what might be an ancestral role for StlB and DIF-1, we deleted the *Pvio stlB* gene. The *Pvio stlB^−^* mutant differed considerably from the *Ddis stlB^−^* mutant. Instead of thinner stalks, *Pvio stlB^−^* fruiting bodies had thicker stalks and overexpressed the stalk gene *ecmAB1*. The stalks were also irregularly shaped and *stlB^−^* fruiting bodies showed reduced formation of whorls of side branches and a reduced number of spores. Expression of a (pre)spore gene was also reduced in *stlB^−^* sorogens. However, it is not clear whether this is due to the StlB product being directly required for spore gene expression, or to the defective formation of secondary branches, where spore maturation normally initiates during *Pvio* fruiting body formation. The overproduction of stalk cells in *stlB^−^* will additionally reduce the number of cells available for spore formation.

All defects of the *Pvio stlB^−^* mutant could be restored by including DIF-1 in the supporting agar, which supports the earlier findings that *Pvio* probably synthesizes DIF-1 (Kay et al. 1992). However, while required for proper stalk morphology, here DIF-1 is not required for actual stalk cell differentiation.

## Supplementary Material

evaa079_Supplementary_Data

## Notes

**Data deposition:** The raw RNA-sequencing reads generated during this study are available in the European Nucleotide Archive under study number PRJEB34080. The processed RNA-Seq data set is listed as supplementary data Pvio_Celltype_TPM.xlxs, Supplementary Material online. The *Polysphondylium violaceum* Whole Genome Shotgun project has been deposited at DDBJ/ENA/GenBank under the accession AJWJ00000000. The version described in this article is version AJWJ01000000.
