# Supplementary material for: Loss of the Polyketide Synthase StlB Results in Stalk Cell Overproduction in Polysphondylium violaceum
Source: Genome Biol Evol. 2020 Apr 18;12(5):674–83. doi: 10.1093/gbe/evaa079 (PMC7259674; doi:10.1093/gbe/evaa079)
Supplement: evaa079_Supplementary_Data [file evaa079_supplementary_data.zip › NaritaSupplementalinfo_v1.pdf]

SUPPLEMENTAL FIGURES AND TABLES

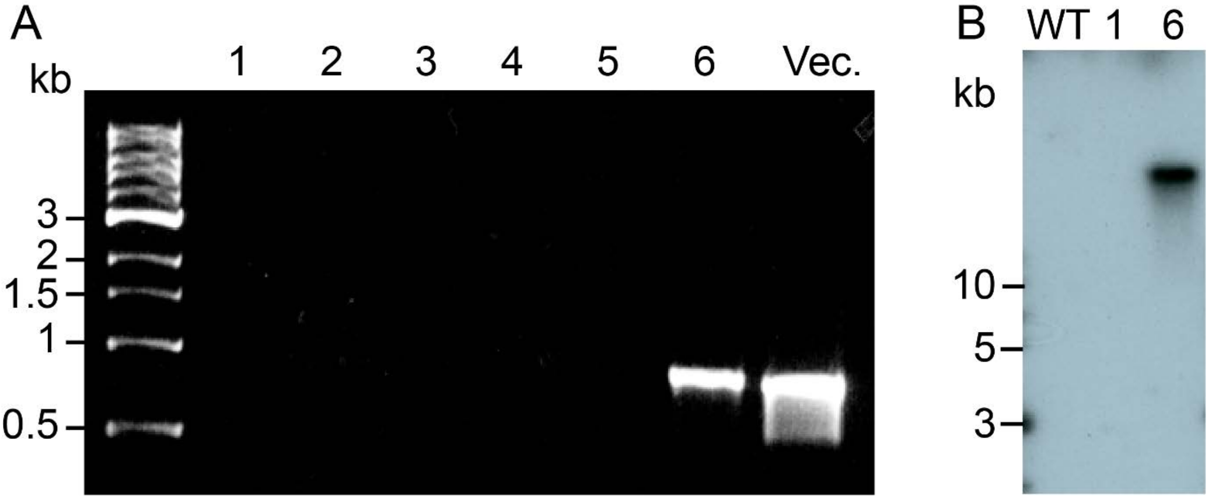

Figure S1. Confirmation of *Pvio* transformation.

A. *PCR*. Genomic DNAs were isolated from six apparently G418 resistant clones from an initial pilot experiment to transform *Pvio* with vector pA15-gal, and were subjected to PCR amplification with primers LacZ-5 and LacZ-3 (Table S1), which amplify a 0.9 kb *lacZ* fragment in the pA15-gal vector, Only 1 of the 6 tested clone (6) appeared to be transformed.

B. *Southern blot analysis*. Genomic DNAs of 2 clones (1 and 6) and untransformed cells (WT) were digested *Xba*I and Southern blots were probed with a ^{32}PdATP-labeled G418 resistant gene fragment, which was generated by digestion of pA15-Gal with PstI. Only a single band was detected in clone 6, which indicated that only one copy of vector was inserted in the genome DNA. Because pA15-Gal has a single *Xba*I site, outside its *LacZ* gene), if two or more copies of the vector had inserted, at least two bands would be detected.

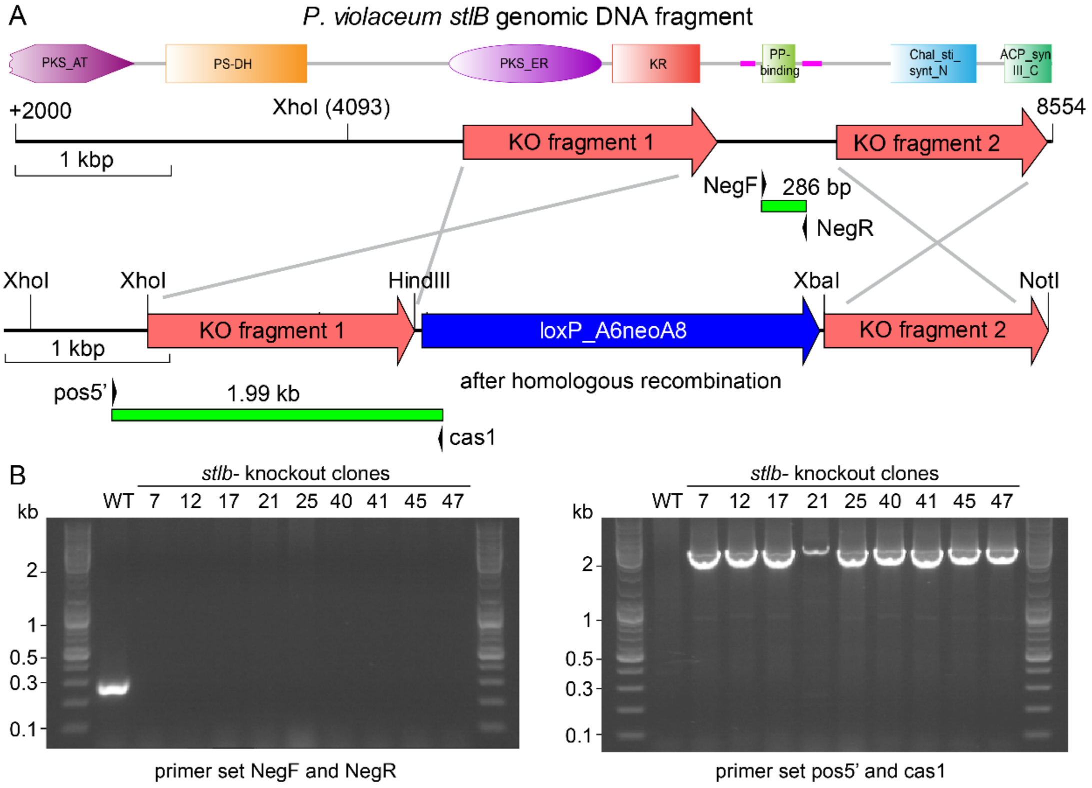

Figure S2. ***StlB***$^{-}$ **KO construct and diagnosis**

A. KO construct. A *Pvio stlB* knock-out construct was designed to replace the essential phospho-pantetheine (PP) binding domain of *stlB* with the LoxP-Neo cassette after successful homologous recombination. The positions of the oligonucleotide primers and sizes of the amplified products that signify absence (neg) or presence (pos) of homologous recombination are indicated.

B. *PCR diagnosis.* Genomic DNAs of 9 out of 27 tested G418 resistant clones that were likely candidates for homologous recombination as judged from a pilot PCR test with primer pair NegF/NegR were retested together with wild-type (WT) cells with primer pairs NegF/NegR and pos5'/cas1 respectively. All clones also tested positive with primer pair pos5'/cas1.

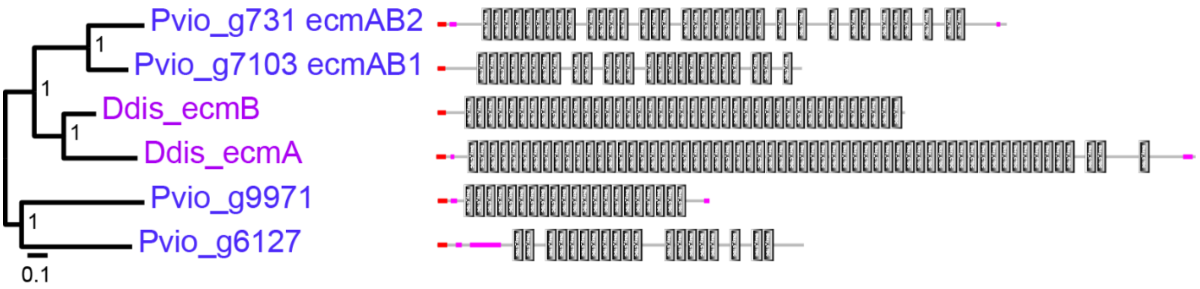

Figure S3. ***Pvio ecmA*** **and** ***ecmB*****-like genes**

Homologs of *Ddis ecmA* and *ecmB* were identified by Blast search of the *Pvio* transcriptome. Protein sequences of the four closest hits were aligned and subjected to Bayesian phylogenetic inference. All proteins mostly consist of 24 amino-acid repeats that were annotated as Dicty_CTDC domains. *Ddis ecmA* and *ecmB* appear to be the result of a recent gene duplication and a similar duplication occurred in *Pvio*. There are therefore no clear *Pvio ecmA* or *ecmB* orthologs and we named the *Pvio* paralogs *ecmAB1* and *ecmAB2*

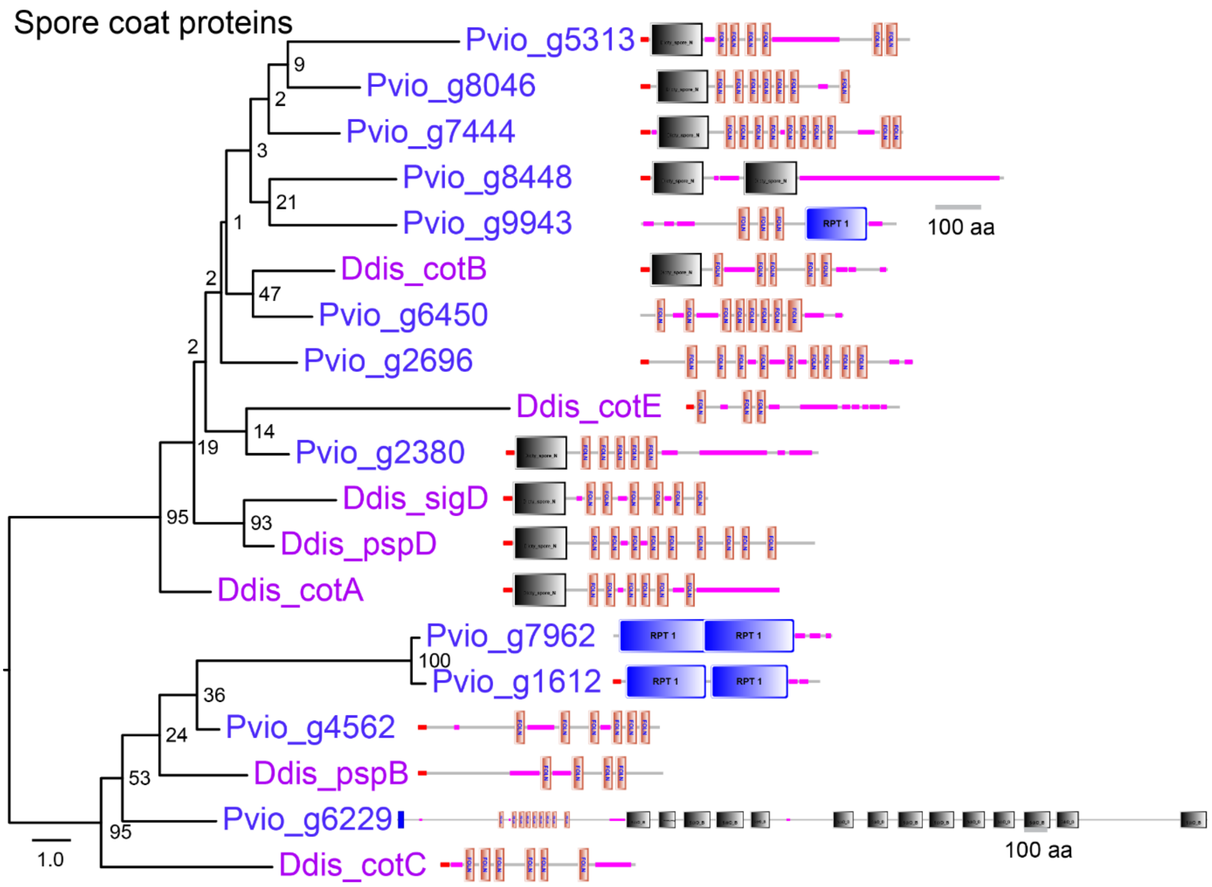

Figure S4. ***Pvio*** **putative sporecoat genes**

Homologs of a range of *Ddis* spore-coat proteins were identified by Blast search of the *Pvio* transcriptome. Protein sequences of the closest hits were aligned and subjected to phylogenetic inference by RAXML. The tree was poorly resolved as evident by the low bootstrap support at the nodes. The tree was annotated with the protein domain architecture of the proteins. The spore coat genes mostly underwent species-specific gene duplications, which makes it difficult to assign orthologs.

Table S1. Oligonucleotide primers used in this work

Name	DNA sequence
Pv_stlB_Frag1F	ATACTCGAGGACAATCACCCAAAGCCAATCAAG
Pv_stlB_Frag1R	ATAAAGCTTTGTTTAAGAGATGGATAAGGAGTG
Pv_stlB_Frag2F	ATATCTAGATTTAGTTACGCCTACAGCGATGC
Pv_stlB_Frag2R	ATAGCGGCCGCCAATACTAGCACCTGGTGAAAAGG
Pv_stlB_NegF	CAAGCAATATGGACACTGCTCAAG
Pv_stlB_NegR	GACTTACTGGTCTTAAAGGTGGAG
Pv_stlB_Pos5'	TACTCTTACCAATGAAATGCACTC
cas1	GGGCAAATCTGTAATTTTCAG
Pv_ecmAB1_P F	ATATCTAGATATACAACCCCCAACTTGAATTTC
Pv_ecmAB1_P R	ATAAGATCTCATTCTTTAATAAATAATTGGAGAGAGAAG
LacZ-5	ACCGTCACGAGCATCATCCT
LacZ-3	AGCGACATCCAGAGGCACTT
Pv-g1612-51	TATTGGTGGTGTCAAAGG
Pv-g1612-31	GAGGTTGTTGTTGGCTTC
Pv-ecmAB1-51	TGCCTGTACTCGTGATTC
Pv-ecmAB1-31	ACAGCTGATTGGGATATG
Pv-rpb5-51	ATGTCTTCTTTCCCACTG
Pv-rpb5-31	GGGTGATGTTTTTAGCTTG

Table S2. Putative *Pvio* spore coat genes

Pvio QSvi11 gene_id	Transcripts per million							
	PVveg1	PVveg2	PVstalk1	PVstalk3	PVstalk4	PVspore1	PVspore2	PVspore3
g1612.t1	0.9	0.9	3.8	0.2	2.8	221.2	165.7	34.6
g7962.t1	1.9	1.0	1.5	0.1	2.1	56.6	36.2	5.9
g4562.t1	0.0	0.0	3.3	0.9	30.8	1.7	2.2	1.9
g6229.t1	2.3	2.1	0.4	0.5	1.1	0.0	0.0	0.0
g2380.t1	0.1	0.2	6.9	1.2	51.4	1.6	2.0	0.6
g2696.t1	0.0	0.0	1.4	1.1	16.2	1.2	2.6	1.1
g6450.t1	0.0	0.0	3.0	0.5	26.9	0.6	1.3	0.3
g9943.t1	0.8	0.5	9.9	1.3	99.3	4.4	2.2	0.9
g8448.t1	4.8	4.8	2.5	2.2	22.4	0.7	0.7	0.8
g7444.t1	0.2	0.0	68.9	16.8	242.7	2.9	2.6	0.6
g8046.t1	0.3	0.4	6.0	1.3	11.3	9.5	6.3	9.9
g5313.t1	1.1	1.2	8.4	6.8	8.9	0.1	0.2	0.1

Normalized transcript reads for the *Pvio* homologs of *Ddis* spore coat genes, shown in figure S4 were retrieved from supplemental data file Pvio_Celltype_TPM.xlsx.
